# Beneficial Effect of Left Ventricular Remodeling after Early Change of Sacubitril/Valsartan in Patients with Nonischemic Dilated Cardiomyopathy

**DOI:** 10.3390/medicina57050416

**Published:** 2021-04-25

**Authors:** Hyue-Mee Kim, Kyung-Hee Kim, Jin-Sik Park, Byung-Hee Oh

**Affiliations:** 1 Division of Cardiology, Department of Internal Medicine, Chung-Ang University Hospital, Seoul 06973, Korea; chemie27@naver.com; 2 Division of Cardiology, Heart Stroke Vascular Center, Incheon Sejong Hospital, 20, Gyeyangmunhwa-ro, Gyeyang-gu, Incheon 21080, Korea; pjsheart@sejongh.co.kr (J.-S.P.); ohbhmed@sejongh.co.kr (B.-H.O.)

**Keywords:** sacubitril/valsartan, nonischemic dilated cardiomyopathy, left ventricular remodeling, heart failure

## Abstract

*Background and Objectives:* Evidence for effectiveness of early change from angiotensin II receptor blockers (ARBs) or angiotensin-converting enzyme inhibitors (ACEIs) to sacubitril/valsartan is lacking. We aimed to investigate whether early changes to sacubitril/valsartan could improve outcomes in patients with nonischemic dilated cardiomyopathy (DCM) in real-world practice. *Materials and Methods*: A total of 296 patients with nonischemic DCM who were treated with ARB or ACEI continuously (group A, *n* = 150) or had their medication switched to sacubitril/valsartan (group S, *n* = 146) were included. The sacubitril/valsartan group was divided into early change (within 60 days, group S/E, *n* = 59) and late change (group S/L, *n* = 87) groups. Changes in echocardiographic parameters from the time of initial diagnosis to the last follow-up were analyzed. *Results*: Patients in group S showed greater left ventricular (LV) end-diastolic dimension (EDD) (group A vs. S, 61.7 ± 7.4 vs. 66.5 ± 8.0, *p* < 0.001) and lower LV ejection fraction (LVEF) (28.9 ± 8.2% vs. 23.9 ± 7.5%, *p* < 0.001) than those in group A at initial diagnosis. During a median follow-up of 76 months, patients in group S/E, ∆ LVEF (%) and ∆ LVESD (mm) were significantly improved compared with those in patients in group A (group A vs. S/E, ∆ LVEF, *p* = 0.036; ∆ LVESD, *p* = 0.023) or S/L (group S/E vs. S/L, ∆ LVEF, *p* = 0.05; ∆ LVESD, *p* = 0.005). Among patients whose medications were switched to sacubitril/valsartan, those with an earlier change showed a significant correlation with greater LVEF improvement (r = −0.367, *p* < 0.001) and LV reverse remodeling (r = 0.277, *p* < 0.001). *Conclusions*: in patients with nonischemic DCM, an early switch to sacubitril/valsartan was associated with greater improvement in LV function. Patients might benefit in terms of LV function by early switching to sacubitril/valsartan.

## 1. Introduction

Heart failure (HF) is the most rapidly growing cardiovascular disease and constitutes a major part of the global disease burden. It is a chronic condition with intermittent acute events that leads to high mortality, frequent hospitalization, and poor quality of life [1,2,3]. For patients diagnosed with HF with reduced ejection fraction (HFrEF), pharmacologically blocking the renin-angiotensin-aldosterone pathways and adrenergic system is known to be effective [4]. Despite the development of several agents for blocking these pathways, the mortality and morbidity associated with HF remain high [4,5]. Recently, new agents acting on the renin-angiotensin-aldosterone and neutral endopeptidase systems have demonstrated greater improvement in functional class and reduction in cardiac mortality compared with that seen the angiotensin-converting enzyme inhibitor (ACEI), enalapril, in patients with HFrEF [6]. The subgroup analysis in the PARADIGM-HF study and several other studies have suggested that sacubitril/valsartan improves left ventricular (LV) function and reduces hospitalization and cardiac mortality rates [7,8,9,10,11]. However, most of the subsequent studies on LV function had short follow-up duration or were single-arm studies. The subgroup analysis in the PARADIGM-HF study reported pronounced changes in the N-terminal pro B-type natriuretic peptide (NT-proBNP) level with early switch to sacubitril/valsartan from enalapril [12,13]. However, the current guidelines recommend replacing ACEI or angiotensin receptor blockers (ARB) with sacubitril/valsartan only if HF symptoms persist despite optimally blocking the three pathways [4]. Therefore, we aimed to investigate whether early switch to sacubitril/valsartan from ACEI or ARB could be more effective in improving the LV function in patients with HFrEF, especially in patients with nonischemic dilated cardiomyopathy (DCM).

## 2. Methods

### 2.1. Study Population

We retrospectively analyzed the medical records of patients diagnosed with nonischemic DCM between January 2009 and November 2019. We included 150 consecutive patients with HFrEF who were continuously prescribed ACEI/ARB (group A) and 146 patients whose prescription had been switched to sacubitril/valsartan from ACEI/ARB (group S) from the HF database in Mediplex Sejong Hospital. The sacubitril/valsartan group was divided based on early change to sacubitril/valsartan (within 60 days; group S/E, *n* = 59) or late change (group S/L, *n* = 87). HF due to nonischemic DCM was diagnosed based on echocardiographic, clinical, and laboratory findings. Nonischemic DCM is defined as dilation of LV chamber and LV ejection fraction (LVEF) of less than 35%. All included patients underwent coronary angiography or coronary computed tomography, and all of them did not meet Felker criteria [14] of ischemic cardiomyopathy. Patients who were younger than 18 years, those who had combined significant valvular heart disease, or those who had undergone cardiac resynchronization therapy were excluded. Responders to HF medication were defined as patients with an increase in LVEF from ≥10% to a final value of ≥35% according to previous studies [10,15]. The present study was carried out according to the principles of the Declaration of Helsinki and was approved by the Clinical Research Institute of Mediplex Sejong Hospital (approved on 9 June 2020; IRB No. 2011).

### 2.2. Transthoracic Echocardiography and Electrocardiography

Echocardiographic examinations were performed at the time of initial diagnosis and at the last follow-up using commercially available equipment (Vivid 7, GE Medical System, Horten, Norway, or E9, Philips Medical Systems, Andover, MA, USA). All patients underwent conventional two-dimensional, M-mode, and color Doppler ultrasonography in accordance with the American Society of Echocardiography guidelines [16]. LV end-diastolic dimension (LVEDD), LV end-systolic dimension (LVESD), and wall thickness were obtained using M-mode or two-dimensional images. The LV end-diastolic and end-systolic volumes were calculated from the apical two-chamber and four-chamber views and LVEF was measured using the Simpson’s biplane method. Left atrial (LA) volumes were determined using the biplane area-length method at end-ventricular systole and LA volume index was calculated as LA volume divided by the body surface area. Right ventricular systolic pressure was estimated from the peak velocity of tricuspid regurgitation with right atrial pressure.

### 2.3. Outcomes

Patients were followed up and their clinical records were reviewed until February 2020. The primary outcomes were difference in LVEF and degree of LV reverse remodeling between the initial echocardiogram and the one acquired at the final follow-up in the two groups. Additionally, the association between the duration from the initial diagnosis to the switch to sacubitril/valsartan administration and the degree of LVEF improvement and LV reverse remodeling were analyzed. Hospitalization for HF and cardiac death were recorded to assess the secondary outcomes.

### 2.4. Statistical Analyses

Continuous variables were expressed as mean ± standard deviation values, and categorical variables were expressed as numbers and percentages. Comparisons between the groups were performed using a Student’s *t*-test or Mann-Whitney U test. A Chi-squared test or Fisher’s exact test was used for categorical variables. Changes in the echocardiographic parameters from the initial diagnosis to the last follow-up were compared using Student’s *t*-test or the repeated measures analysis of variance test. The correlation between the duration from the initial diagnosis to the switch to sacubitril/valsartan and the echocardiographic parameters were analyzed using Pearson’s correlation coefficient. Event rates were estimated using event counts and exposures over time. Univariate Cox proportional hazards regression analyses were performed to evaluate the predictive value of each variable. Significant variables were introduced into a multivariate Cox proportional hazards regression model. Hazard ratios (HR) and 95% confidence intervals were calculated. Event-free survival analyses were conducted using the Kaplan-Meier method with a log-rank test. All statistical analyses were performed using IBM SPSS Statistics 22.0 (IBM Corp., Armonk, NY, USA) and a *p*-value of <0.05 was considered statistically significant.

## 3. Results

### 3.1. Baseline Characteristics

The baseline characteristics of the patient groups according to medications are depicted in Table 1. The median follow-up after initial diagnosis was 714 days (interquartile range, 388–1334 days) for patients who switched to sacubitril/valsartan and 1034 days (interquartile range, 631–1930 days) for patients who continued with ACEI/ARB. Patients switched from ACEI/ARB to sacubitril/valsartan at a median duration of 236 days (interquartile range: 57.8–810 days) after the initial diagnosis. The mean age of patients with nonischemic DCM patients was 58.8 ± 16.0 years and 212 (71.6%) patients were men. Patients who switched to sacubitril/valsartan were younger than those who continued with ACEI/ARB (61.1 ± 14.9 years in group A vs. 56.7 ± 16.9 years in group S, *p* = 0.029). There were no significant differences in the prevalence of hypertension, diabetes mellitus, stroke, chronic kidney disease, and coronary artery disease between patients who continued with ACEI/ARB, those with early change to sacubitril/valsartan, and those with late change. Laboratory findings including NT-proBNP levels were not significantly different, but the estimated glomerular filtration rate was higher in patients who continued with ACEI/ARB than those who switched to sacubitril/valsartan. There were no differences in the cardiovascular medications including spironolactone and ivabradine, but a slightly higher use of beta-blockers was observed in patients who switched to sacubitril/valsartan.

### 3.2. Echocardiographic Changes from the Initial Diagnosis to the Last Follow-Up

The initial echocardiographic parameters are summarized in Table 2. The LV wall thickness, ratio between early mitral inflow velocity and mitral annular early diastolic velocity (E/e’ ratio), pulmonary artery systolic pressure, LA dimension, and LA volume index were similar between patients who continued with ACEI/ARB, those with early change to sacubitril/valsartan, and those with late change. However, patients on sacubitril/valsartan had greater LVEDD (group A vs. S, 61.7 ± 7.4 vs. 66.5 ± 8.0 mm, *p* < 0.001), greater LVESD (group A vs. S, 51.3 ± 8.7 vs. 57.6 ± 9.5 mm, *p* < 0.001), and lower LVEF (group A vs. S, 28.9 ± 8.2% vs 23.9 ± 7.5%, *p* < 0.001) than patients who continued with ACEI/ARB did (group A). No significant changes were observed between patients in the early change (group S/E) and late change (group S/L) groups, except for LVEDD.

The median duration from the initial to last echocardiography was 559 days (interquartile range: 336–1083 days) for patients who switched to sacubitril/valsartan (group S) and 702 days (interquartile range: 324–1524 days) for patients who continued with ACEI/ARB (group A). In echocardiographic evaluation during the follow-up, LV reverse modeling and recovery of LVEF were observed in all patient groups. In patients who continued with ACEI/ARB (group A), LVEDD decreased from 61.7 ± 7.4 to 56.6 ± 7.2 mm (*p* < 0.001) and LVEF increased from 28.9 ± 8.2% to 42.3 ± 11.3% (*p* < 0.001). Similarly, LVEDD diminished from 66.5 ± 8.0 to 61.4 ± 9.3 mm (*p* < 0.001) in patients who switched to sacubitril/valsartan (group S) and LVEF improved from 23.9 ± 7.5% to 36.2 ± 11.4% (*p* < 0.001). The LA volume index, E/e’ ratio, and estimated pulmonary artery systolic pressure decreased in both groups (Table 3).

### 3.3. Correlation between the Time from the Initial Diagnosis to the Switch to Sacubitril/Valsartan and the Recovery of LVEF

**Figure 1:**
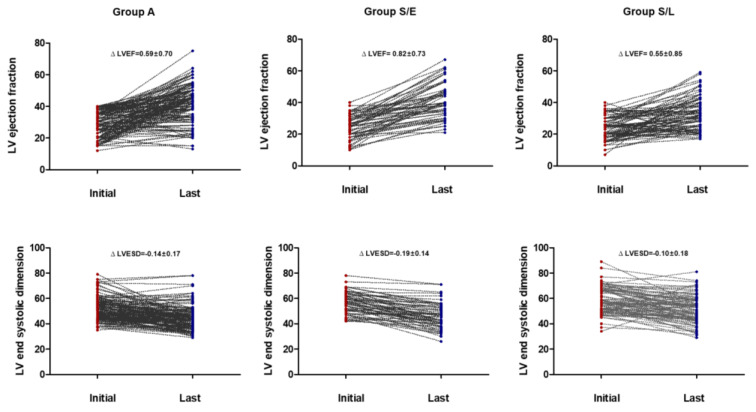
Changes in echocardiographic parameters from the initial diagnosis to the last follow-up.

**Figure 2:**
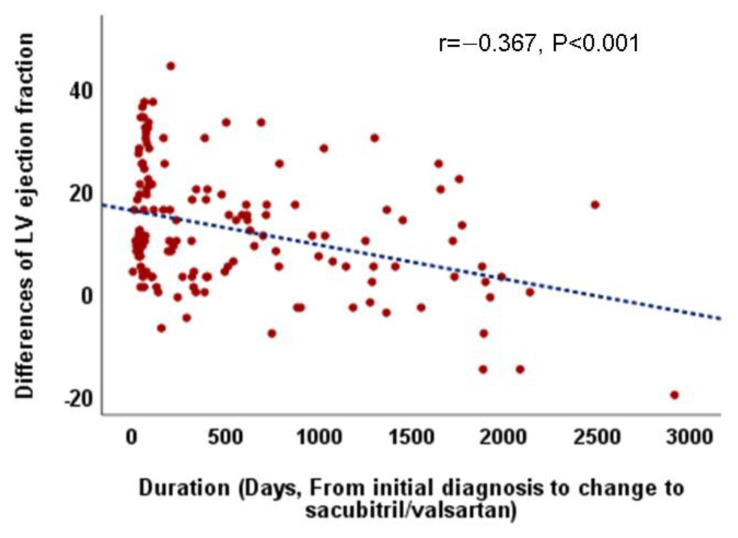
Correlation between the duration from the initial diagnosis to the change to sacubitril/valsartan and echocardiographic parameters especially left ventricular ejection fraction.

**Figure 3:**
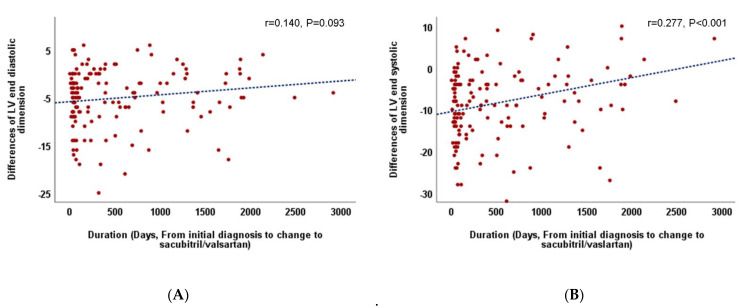
(**A**). Correlation between the duration from the initial diagnosis to the change to sacubitril/valsartan and left ventricular end-diastolic dimension. (**B**). Correlation between the duration from the initial diagnosis to the change to sacubitril/valsartan and left ventricular end-systolic dimension.

Considering the overall follow-up period, there were no differences in LVEF improvement and LV reverse remodeling between patients who continued with ACE/ARB (group A) and those who switched to sacubitril/valsartan (group S). However, in patients who switched to sacubitril/valsartan within 60 days (early change, group S/E), an increase in LVEF and decrease in LVESD were more prominent than those in patients who switched late (S/L) (group S/E vs. S/L, ∆ LVEF, 0.82 ± 0.73 vs. 0.55 ± 0.85, *p* = 0.05, ∆ LVESD, −0.19 ± 0.14 vs. −0.10 ± 0.18, *p* = 0.005) and those who continued with ACEI/ARB (group A) (group A vs. S/E, ∆ LV-EF, 0.59 ± 0.70 vs. 0.82 ± 0.73, *p* = 0.036, ∆ LVESD, −0.14 ± 0.17 vs. −0.19 ± 0.14, *p* = 0.023) (Table 4, Figure 1). Additionally, the relationship between the time from the initial diagnosis to the switch to sacubitril/valsartan and the improvement in LVEF (r = −0.367, *p* < 0.001, Figure 2) and decrement in LVESD (r = 0.277, *p* < 0.001, Figure 3B) showed a significant correlation. Figure 2 shows the correlation between the time from the initial diagnosis to the switch to sacubitril/valsartan and the recovery of LVEF and LV reverse remodeling.

### 3.4. Clinical Outcomes

During the follow-up, 93 patients were admitted for HF aggravation, and cardiac death was observed in three patients. All deaths occurred after admission for HF exacerbation. The overall incidences of hospitalization for HF aggravation and for cardiac death were 10.0 and 0.32 per 100 person-years, respectively. No significant differences in cardiac events were observed between the groups during the entire follow-up (10.7/100 person-years in group A vs. 9.64/100 person-years in group B, log-rank *p* = 0.794). In the univariate analysis, age (HR, 1.028; *p* < 0.001), presence of hypertension (HR, 1.838; *p* = 0.008), chronic kidney disease (HR, 2.162; *p* = 0.003), atrial fibrillation (AF) (HR,2.030; *p* = 0.001), no prescription of beta-blockers (HR, 2.696; *p* = 0.002), dilated LA (HR 1.010; *p* < 0.001), and non-response to HF medication (HR, 2.213; *p* < 0.001) were significantly associated with hospitalization due to HF during the follow-up. However, LVEF and LVEDD were not significant determinants of hospitalization for HF in patients with HFrEF due to nonischemic DCM (Table 5). In the multivariate analysis, the absence of beta-blockers (HR, 3.144; *p* = 0.001), nonresponse to HF medication (HR, 1.887; *p* = 0.016), presence of AF (HR, 1.945; *p* = 0.006), and dilated LA (HR, 1.010; *p* = 0.020) were significant predictors of HF admission.

## 4. Discussion

In the present study, we demonstrated that guideline-directed medical therapy and blocking the renin-angiotensin-aldosterone pathway and adrenergic system in patients with HFrEF due to nonischemic DCM resulted in improved LV function and decreased cardiac chamber. In patients with worsened LV function, larger heart size, and limited response to ACEI/ARB, sacubitril/valsartan showed similar effects in terms of improvement in LVEF and LV reverse remodeling. Additionally, an earlier change to sacubitril/valsartan was associated with greater improvement in LV function in patients with nonischemic HFrEF. These findings suggest that sacubitril/valsartan could be helpful in improving the outcomes in patients with non-ischemic DCM who have reduced EF and greater improvement could be observed with earlier administration of sacubitril/valsartan.

LV reverse remodeling and improved LVEF are one of the most important outcomes in clinical practice while treating patients with dilated LV and reduced LVEF. In addition, they are associated with a good prognosis in most types of HF [17]. Several studies have reported cardiac reverse remodeling and improvement in LVEF with sacubitril/valsartan. Most of these studies had short follow-up periods or follow-up in a single group without comparison with previous ACEI/ARB [7,18,19]. In the present long-term follow-up study, both sacubitril/valsartan and ACEI/ARB groups showed a decrease in LVEDD and improvement in LVEF. Although patients who switched to sacubitril/valsartan had larger cardiac chambers and a greater decrease in LV function, improvement of LV function tended to be more prominent in patients who early switched to sacubitril/valsartan after diagnosis than in those who continued with ACEI/ARB. Additionally, a significant correlation was observed between the time from the initial diagnosis to the switch to sacubitril/valsartan and the improvement in LVEF. Due to the small number of patients who had an early switch to sacubitril/valsartan, differences in clinical outcomes such as hospitalization due to HF and cardiac death according to the time to sacubitril/valsartan switch could not be demonstrated. However, previous studies have reported that improvements in LVEF and NT-proBNP levels were associated with a decrease in HF hospitalization and cardiac outcome [17,20]. A recent study reported that NT-proBNP levels were highly decreased in patients with early switch to sacubitril/valsartan from enalapril [11]. Our study showed similar results for the early switch to sacubitril/valsartan. Taken together, an early switch to sacubitril/valsartan could be more effective and could be associated with a good prognosis in patients with HFrEF.

In our study, improvement in LV function and LV reverse remodeling during the entire follow-up period were prominent in group S/E compared to in group S/L. This finding might be related to the characteristics of nonischemic DCM and the treatment guidelines for HF [4]. There were several cases in which the switch from ACEI/ARB to sacubitril/valsartan was made after a considerably long time following the diagnosis of DCM. According to the current HF guidelines, the insurance standard for sacubitril/valsartan in Korea is applicable only in cases where the LV function is less than 40% and the dyspnea levels are above NYHA class II, even with the use of ACEI/ARB, beta-blockers, and spironolactone in patients with HFrEF. Therefore, some HFrEF patients who did not meet that criteria could not switch to sacubitril/valsartan easily and were able to use sacubitil/valsartan after HF worsened. Additionally, some of patients in group S/L were not responsive to previous ACEI/ARB treatment and showed dyspnea levels above New York Heart Association (NYHA) class II despite using ACEI/ARB and other HF medications for a long time. In clinical practice, if patients with nonischemic DCM do not show early improvement in LV function after initiation of HF medication (<6 months), the improvement rates of LV reverse remodeling and LVEF generally decrease, which is associated with poor prognosis [15]. Thus, a relatively high proportion of patients who did not respond to ACEI/ARB and BB for long periods in group S/L might affect the outcomes. During the follow-up, LVEDD and LVESD decreased and LVEF improved in both groups. In group S, patients with an earlier switch to sacubitril/valsartan showed greater LVEF improvement and a greater decrease in LVESD, but no marked decrease in LVEDD. Thus, it is likely that the considerable improvement in LV function in patients with early switch to sacubitril/valsartan compared with that in patients with late switch was due to the greater degree of decrease in LVESD rather than the decrease in LVEDD. The underlying mechanism for the greater effect of decreased LVESD on the improvement in LVEF when compared with the effect of LVEDD is unclear. Since the natriuretic peptide (NP) system enhances vasodilation and decreases afterload [21], the aforementioned results may be related to the enhancement of the NP system by sacubitril/valsartan. This issue requires further investigation with a larger sample size in a prospective setting.

The overall incidence of HF admission in nonischemic DCM was 10.0/100 person-years. Considering their predictive value for hospitalization due to HF, LVEDD, and LVEF did not have a significant impact on hospitalization due to HF. However, the nonresponse to HF medication, presence of AF, and absence of beta-blockers showed superior predictive value for cardiac events. Since the patients in our study had severe LV dysfunction and most of them received guideline-directed medical therapy, the management of accompanying AF requires more attention. Currently, the methods of cardiac resynchronization therapy, heart transplantation, and LV assist devices for patients who do not respond to drugs are used. However, since it is difficult to apply it to all patients, more studies on improving the prognosis of nonresponders should be carried out.

The present study had several limitations. This was a retrospective single-center study. Therefore, the decision regarding the use of sacubitril/valsartan was not randomized. Considering the HF guidelines and the insurance standards in Korea, patients who switched to sacubitril/valsartan had more severe disease and used more types of HF medications. The timing of the follow-up echocardiography was different for the groups. However, the response to medication therapy in HFrEF patients showed a marked improvement until approximately 1 year and then showed a plateau phase [22]. Due to the small sample size, the results of the present study should be interpreted with caution. Randomized trials with larger sample sizes are required to confirm these results.

## 5. Conclusions

An earlier switch to sacubitril/valsartan was significantly associated with a greater improvement in LV function in patients with nonischemic DCM. Early switch to sacubitril/valsartan might be helpful in improving the outcomes of patients with HFrEF.

## Figures and Tables

**Table 1:** Baseline characteristics according to the groups.

	ACEI/ARBGroup A (*n* = 150)	Early Change to sacubitril/Valsartan Group S/E (*n* = 59)	Late Change to Sacubitril/Valsartan Group S/L (*n* = 87)	*p* Value †	*p* Value *
Age (years)	61.1 ± 14.9	55.4 ± 18.6	57.6 ± 15.8	0.029	0.453
Male (*n*, %)	106 (69.3%)	45 (76.3%)	64 (73.6%)	0.270	0.711
**Underlying diseases (*n*, %)**					
Hypertension	51 (33.3%)	16 (27.1%)	24 (27.6%)	0.437	0.951
Diabetes mellitus	40 (26.1%)	15 (25.4%)	20 (23.0%)	0.932	0.736
Stroke	5 (3.3%)	2 (3.4%)	4 (4.6%)	0.984	0.719
Chronic kidney disease	21 (13.7%)	6 (10.2%)	15 (17.2%)	0.458	0.234
Coronary artery disease	4 (2.6%)	4 (6.8%)	3 (3.4%)	0.164	0.357
**Medication**					
ACEI/ARB	142 (94.7%)	59 (100.0%)	86 (98.9%)	0.071	0.410
Beta blocker	135 (90.0%)	58 (98.3%)	83 (95.4%)	0.042	0.346
Spironolactone	113 (75.3%)	48 (81.4%)	65 (74.7%)	0.353	0.348
Ivabradine	13 (8.7%)	5 (8.5%)	13 (14.9%)	0.965	0.243
**Sacubitril/Valsartan**					
Initiation at outpatient clinic (*n*,%)	-	55 (93.2%)	79 (90.8%)	-	0.602
Starting dose (mg/day)	-	126.3 ± 84.3	133.9 ± 83.4	-	0.590
Last maintenance dose (mg/day)	-	184.8 ± 111.1	193.1 ± 110.8	-	0.656
Achievement of target dose (*n*,%)	-	10 (16.9%)	16 (18.4%)	-	0.823
**Laboratory examination**					
Hemoglobin (g/dL)	13.8 ± 2.0	14.1 ± 1.9	13.5 ± 2.5	0.305	0.116
Blood urea nitrogen (mg/dL)	19.6 ± 10.1	19.2 ± 8.7	19.4 ± 7.9	0.698	0.884
Creatinine (mg/dL)	1.15 ± 0.8	1.0 ± 0.3	1.0 ± 0.3	0.115	0.267
Estimated glomerular filtration rate (mL/min/1.73 m^2^)	73.4 ± 24.2	82.4 ± 21.4	79.4 ± 21.2	0.018	0.413
Sodium (mEq/L)	136.0 ± 22.8	140.1 ± 3.4	139.3 ± 3.2	0.168	0.140
Potassium (mEq/L)	4.2 ± 0.6	4.3 ± 0.6	4.3 ± 0.5	0.582	0.824
BNP (pg/mL)	594.0 (194.0–1179.5)	931.0 (478.3–2194.0)	842.5 (460.5–2018.6)	0.094	0.991
Pro-BNP (pg/mL)	1931.0 (792.8–4226.0)	1070.0 (283.0–5898.0)	1786.0 (1101.5–3569.8)	0.466	0.358

ACEI: angiotensin-converting enzyme inhibitor, ARB: angiotensin receptor blocker, BNP: B-type natriuretic peptide, S/E: sacubitril/valsartan early change, S/L: sacubitril/valsartan late change. † *p* value for differences between groups A and S/E, * *p* value for differences between groups S/E and S/L.

**Table 2:** Baseline echocardiographic and electrocardiographic exams according to the groups.

	ACEI/ARBGroup A (*n* = 150)	Early Change to Sacubitril/Valsartan Group S/E (*n* = 59)	Late Change to Sacubitril/Valsartan Group S/L (*n* = 87)	*p* Value †	*p* Value *
**Echocardiographic exam**					
LVEDD	61.7 ± 7.4	64.8 ± 6.6	67.6 ± 8.8	0.004	0.028
LVESD	51.3 ± 8.7	56.7 ± 8.4	58.3 ± 10.2	<0.001	0.292
Mean LV wall thickness	10.3 ± 6.7	9.4 ± 1.5	9.6 ± 1.6	0.329	0.435
LVEF	28.9 ± 8.2	24.6 ± 7.5	23.5 ± 7.5	0.001	0.381
Septal E/e’	18.9 ± 12.6	19.6 ± 9.4	20.5 ± 12.8	0.797	0.660
LA volume index	54.7 ± 20.4	54.5 ± 20.9	60.7 ± 28.3	0.906	0.192
Pulmonary artery systolic pressure	37.3 ± 15.0	36.4 ± 15.9	41.1 ± 16.3	0.688	0.087
**Electrocardiographic exam**					
Atrial fibrillation	52 (34.0%)	12 (20.3%)	22 (25.3%)	0.043	0.488
LBBB	23 (15.0%)	14 (23.7%)	12 (13.8%)	0.152	0.124
RBBB	6 (4.9%)	3 (5.1%)	2 (2.3%)	0.728	0.364

LV: left ventricle, EDD: end-diastolic dimension, ESD: end-systolic dimension, EF: ejection fraction, LA: left atrium, EDV: end-diastolic volume, ESV: end-systolic volume, LBBB: left bundle branch block, RBBB: right bundle branch block. S/E: sacubitril/valsartan early change, S/L: sacubitril/valsartan late change. † *p* value for differences between groups A and S/E, * *p* value for differences between groups S/E and S/L.

**Table 3:** Echocardiographic changes from the initial diagnosis to the last follow-up.

	ACEI/ARB (Group A)		*p* Value †	Sacubitril/Valsartan (Group S)		*p* Value †
	Initial Diagnosis	Last Follow-Up		Initial Diagnosis	Last Follow-Up	
LVEF (%)	28.9 ± 8.2	42.3 ± 11.3	<0.001	23.9 ± 7.5	36.2 ± 11.4	<0.001
LVEDD (mm)	61.7 ± 7.4	56.6 ± 7.2	<0.001	66.5 ± 8.0	61.4 ± 9.3	<0.001
LVESD (mm)	51.3 ± 8.7	43.8 ± 9.2	<0.001	57.6 ± 9.5	49.4 ± 10.9	<0.001
LA volume index	54.7 ± 20.4	47.4 ± 22.6	<0.001	58.5 ± 25.8	47.6 ± 24.5	<0.001
E/e’ ratio	18.9 ± 12.6	13.1 ± 6.0	<0.001	20.1 ± 11.7	13.3 ± 7.0	<0.001
Pulmonary artery systolic pressure	37.3 ± 15.0	28.6 ± 10.4	<0.001	38.9 ± 16.3	28.7 ± 12.2	<0.001

† *p* value for differences between the initial diagnosis and last follow-up within each group. LV: left ventricle, EF: ejection fraction, EDD: end-diastolic dimension, ESD: end-systolic dimension, LA: left atrium.

**Table 4:** Changes in echocardiographic parameters from the initial diagnosis to the last follow-up.

	ACEI/ARB Group A (*n* = 150)	Early Change to Sacubitril/Valsartan Group S/E (*n* = 59)	Late Change to Sacubitril/Valsartan Group S/L (*n* = 87)	*p* Value †	*p* Value *
∆ LVEF (%)	0.59 ± 0.70	0.82 ± 0.73	0.55 ± 0.85	0.036	0.050
∆ LVEDD (mm)	−0.08 ± 0.10	−0.09 ± 0.08	−0.07 ± 0.10	0.359	0.137
∆ LVESD (mm)	−0.14 ± 0.17	−0.19 ± 0.14	−0.10 ± 0.18	0.023	0.005
∆ LA volume index	−0.10 ± 0.34	−0.17 ± 0.43	−0.12 ± 0.39	0.336	0.512

LV: left ventricle, EF: ejection fraction, EDD: end-diastolic dimension, ESD: end-systolic dimension, LA: left atrium. ∆ value for differences between the initial and last follow-up divided by the initial value. † *p* value for differences between groups A and S/E, * *p* value for differences between groups S/E and S/L.

**Table 5:** Univariate and multivariate analyses for cardiac events during entire periods.

	Univariate Analysis			Multivariate Analysis		
	HR	95% CI	*p* Value	HR	95% CI	*p* Value
Age	1.028	1.013–1.044	<0.001	1.012	0.994–1.030	0.180
LVEF	0.997	0.975–1.019	0.760			
LVEDD	0.996	0.971–1.021	0.727			
LA volume index	1.010	1.003–1.018	<0.001	1.010	1.002–1.019	0.020
Hypertension	1.838	1.176–2.874	0.008	1.750	1.012–3.027	0.045
Diabetes mellitus	1.200	0.753–1.911	0.443			
Chronic kidney disease	2.162	1.309–3.573	0.003	1.054	0.570–1.949	0.868
Stroke	0.949	0.233–3.868	0.932			
Nonresponse to HF medication	2.213	1.445–3.390	<0.001	1.887	1.126–3.163	0.016
Early change to sacubitril/valsartan	0.634	0.223–1.805	0.394			
Absence of RAS blocker	1.501	0.550–4.096	0.428			
Absence of beta blocker	2.696	1.425–5.100	0.002	3.144	1.436–6.882	0.004
Absence of spironolactone	0.975	0.598–1.591	0.920			
Atrial fibrillation	2.030	1.339–3.079	0.001	1.945	1.209–3.130	0.006

LV: left ventricle, EF: ejection fraction, EDD: end diastolic dimension, ESD: end systolic dimension, LA: left atrium, HF: heart failure, RAS: renin angiotensin system, HR: hazard ratio, CI: confidence interval.

## Data Availability

The data presented in this study are available on reasonable request from the corresponding author.
